# Polygenic modifiers impact penetrance and expressivity in telomere biology disorders

**DOI:** 10.1172/JCI191107

**Published:** 2025-06-03

**Authors:** Michael Poeschla, Uma P. Arora, Amanda Walne, Lisa J. McReynolds, Marena R. Niewisch, Neelam Giri, Logan P. Zeigler, Alexander Gusev, Mitchell J. Machiela, Hemanth Tummala, Sharon A. Savage, Vijay G. Sankaran

**Affiliations:** 1 Division of Hematology/Oncology, Boston Children’s Hospital and; 2 Department of Pediatric Oncology, Dana-Farber Cancer Institute, Harvard Medical School, Boston, Massachusetts, USA.; 3 Howard Hughes Medical Institute, Boston, Massachusetts, USA.; 4 Broad Institute of MIT and Harvard, Cambridge, Massachusetts, USA.; 5 Harvard/MIT MD-PhD Program, Program in Biomedical Informatics, Boston, Massachusetts, USA.; 6 Genomics and Child Health, Blizard Institute, Queen Mary University of London, Newark Street, London, United Kingdom.; 7 Division of Cancer Epidemiology and Genetics, National Cancer Institute, Rockville, Maryland, USA.; 8 Department of Pediatric Hematology and Oncology, Hannover Medical School, Hannover, Germany.; 9 Cancer Genomics Research Laboratory, Division of Cancer Epidemiology and Genetics, NCI, Frederick National Laboratory for Cancer Research, Leidos Biomedical Research Inc., Bethesda, Maryland, USA.; 10 Division of Population Sciences, Dana-Farber Cancer Institute, Harvard Medical School, Boston, Massachusetts, USA.

**Keywords:** Genetics, Hematology, Stem cells, Bone marrow, Hematopoietic stem cells, Telomeres

## Abstract

**BACKGROUND:**

Telomere biology disorders (TBDs) exhibit incomplete penetrance and variable expressivity, even among individuals harboring the same pathogenic variant. We assessed whether common genetic variants associated with telomere length combine with large-effect variants to impact penetrance and expressivity in TBDs.

**METHODS:**

We constructed polygenic scores (PGS) for telomere length in the UK Biobank to quantify common variant burden and assessed the PGS distribution across patient cohorts and biobanks to determine whether individuals with severe TBD presentations have increased polygenic burden causing short telomeres. We also characterized rare TBD variant carriers in the UK Biobank.

**RESULTS:**

Individuals with TBDs in cohorts enriched for severe pediatric presentations have polygenic scores predictive of short telomeres. In the UK Biobank, we identified carriers of pathogenic TBD variants who were enriched for adult-onset manifestations of TBDs. Unlike individuals in disease cohorts, the PGS of adult carriers did not show a common variant burden for shorter telomeres, consistent with the absence of childhood-onset disease. Notably, TBD variant carriers were enriched for idiopathic pulmonary fibrosis diagnoses and telomere length PGS stratified pulmonary fibrosis risk. Finally, common variants affecting telomere length were enriched in enhancers regulating known TBD genes.

**CONCLUSION:**

Common genetic variants combined with large-effect causal variants to impact clinical manifestations in rare TBDs. These findings offer a framework for understanding phenotypic variability in other presumed monogenic disorders.

**FUNDING:**

This work was supported by NIH grants R01DK103794, R01HL146500, R01CA265726, R01CA292941, and the Howard Hughes Medical Institute.

## Abstract 2

We find that common genetic polymorphisms that alter telomere length in the general population serve as important modifiers of expressivity and penetrance in presumed monogenic telomere biology disorders.

## Introduction

Variable expressivity and incomplete penetrance — related phenomena where individuals with identical genetic variants display variable symptoms or no clinical symptoms at all — remain poorly understood. Deeper insights into the mechanisms underlying expressivity and penetrance are critical for accurate genetic prognostication in clinical medicine (1, 2). While a variety of factors could contribute, one proposed mechanism is common genetic variation modifying the effect of high-impact rare alleles. Supporting this, in complex diseases, polygenic variation has been shown to modify the phenotypic expression of high-impact variants (3–11). Additionally, in presumed monogenic disease, individual loci have been identified that modify risk (12–14). However, in extremely rare monogenic diseases, where pathogenic variants might be assumed to overwhelm any effect of common variation (15), the extent to which genome-wide polygenic variation affects penetrance and expressivity has not been characterized. This, in part, stems from a lack of quantifiable phenotypic variability in rare disease cohorts, as well as from a paucity of individuals harboring these rare and deleterious alleles in population biobanks, making it incredibly challenging to study the role of common variation in such conditions.

To assess the potential effect of polygenic variation on penetrance and expressivity in rare monogenic disease, we focused on dyskeratosis congenita and related telomere biology disorders (TBDs), which exhibit remarkable clinical heterogeneity despite their presumed monogenic nature. The TBDs are extremely rare disorders — dyskeratosis congenita, the archetypal TBD, has an estimated prevalence of approximately 1 in 1,000,000 in the general population — driven by pathogenic germline variants in genes that regulate telomere length, maintenance, structure, and function (16–63). Variants in these genes display striking variable expressivity and incomplete penetrance related to symptom severity, age of onset, and organ involvement. For example, some carriers of known pathogenic variants in TBD-associated genes present with severe childhood-onset bone marrow failure, while others present in adulthood with idiopathic pulmonary fibrosis or liver disease, and others may have no associated clinical presentation at all (51, 64–67). Genetic anticipation, where phenotypes manifest earlier or become more severe in successive generations, has been suggested to play a role in phenotypic variability in families with TBDs, as have the affected gene and mode of inheritance, but these factors cannot explain all the heterogeneity present (16, 68–70).

As impaired regulation of telomere maintenance is causal for the TBDs and the underlying biological mechanisms are relatively well understood, this group of diseases presents a unique opportunity to test the idea of polygenic modification affecting penetrance and expressivity (16, 17, 71, 72). Rare variants in genes that cause TBDs have been identified, and, conversely, common variants that collectively underlie a considerable fraction of population-level variation in telomere length have been identified in diverse cohorts (73–76). This makes it possible to use telomere length, measured in large population cohorts, as a common trait that reflects the same biology underlying TBDs, providing a unique opportunity to study the impact of common variants in extremely rare TBDs. We hypothesized that common genome-wide genetic polymorphisms, which contribute to the interindividual variation in telomere length in the general population, may combine with large-effect monogenic TBD-causal genetic variants to impact variable expressivity and incomplete penetrance in TBDs.

**Figure 1:**
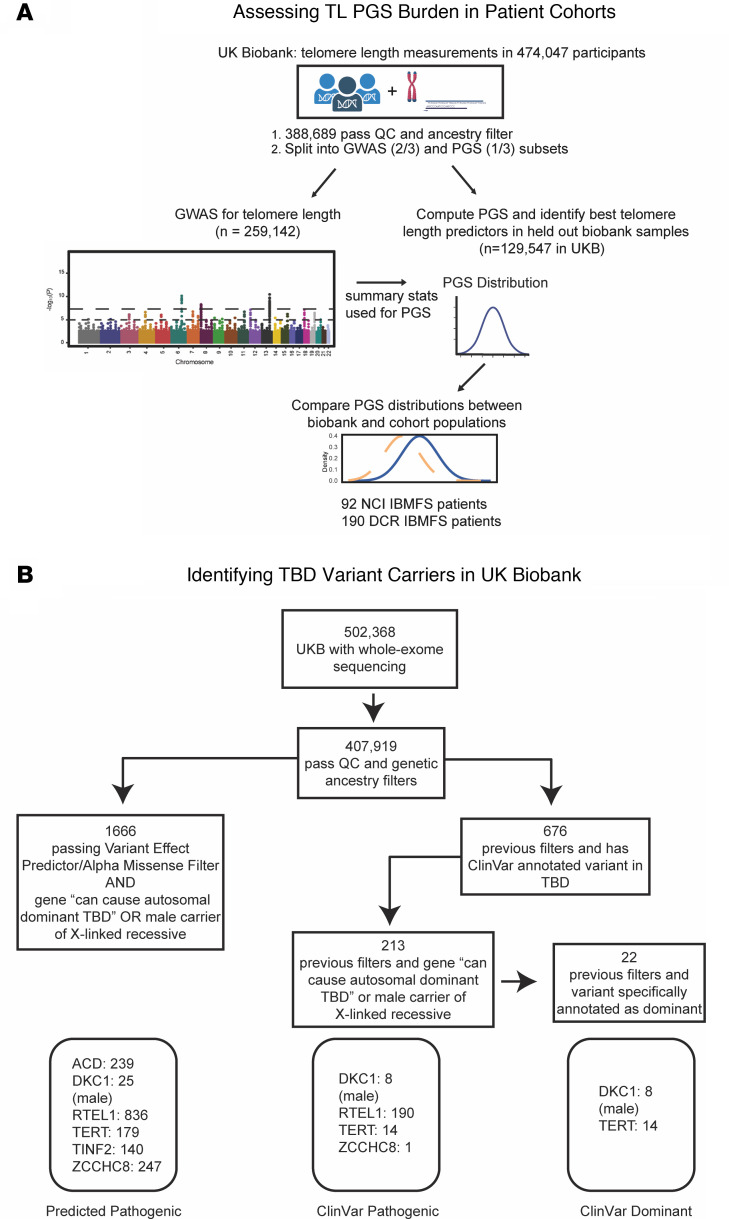
Flow Diagram of Study Design. (**A**) Schematic of study design to assess polygenic variation affecting telomere length across biobank and cohort populations. Individuals with measured telomere length in the UK Biobank were randomly split into GWAS and PGS subsets. A GWAS for telomere length was conducted, and then the best polygenic predictors were identified in the other subset. The resulting PGS represents the common variant burden associated with telomere length. The best PGS was compared between biobank cohorts and disease cohorts. (**B**) Variant carrier ascertainment strategy in UK Biobank, and summary of mutation carriers for each gene in variant carrier groups. Pathogenic variants were alternately defined based on pathogenic prediction using VEP and AlphaMissense, or using ClinVar annotated variants.

Here, we show that common genetic variation across the entire genome contributes to penetrance and expressivity in telomere biology disorders, both in TBD cohorts enriched for individuals presenting with severe childhood-onset bone marrow failure or other hematologic manifestations, as well as in adult biobank cohorts (Figure 1). We further demonstrate that polygenic variation can contribute to variable disease manifestations within a single family with a shared high-impact variant, and that common and rare variation converge on the same biological mechanisms implicated in telomere maintenance. These results provide a framework for exploring the effects of polygenic variation on penetrance and expressivity in rare diseases.

## Results

### Common genetic variation associated with telomere length contributes to TBD variant penetrance and expressivity in inherited bone marrow failure syndrome cohorts.

To assess whether common genetic variation impacts penetrance and expressivity in TBDs, we developed polygenic scores (PGS) using genome-wide common SNPs associated with telomere length (73, 74, 77). For a given individual, these scores provide an estimate of the combined effect of common genetic variants across the genome that increase or decrease telomere length (78, 79). While these common polymorphisms underlie subtle variation in telomere length in the healthy population, we reasoned that the PGS might have a more profound impact in the context of high-impact monogenic alleles that are causal for the TBDs. We therefore applied the most predictive PGS, as determined using PRCise-2 (Supplemental Table 1; supplemental material available online with this article; https://doi.org/10.1172/JCI191107DS1) to the National Cancer Institute (NCI) longitudinal cohort of individuals with inherited bone marrow failure syndromes, including those with TBDs (ClinicalTrials.gov Identifier: NCT00027274). The NCI cohort included 92 patients with dyskeratosis congenita and related TBDs and is significantly enriched for individuals presenting with bone marrow failure or other severe phenotypes (80).

**Figure 2:**
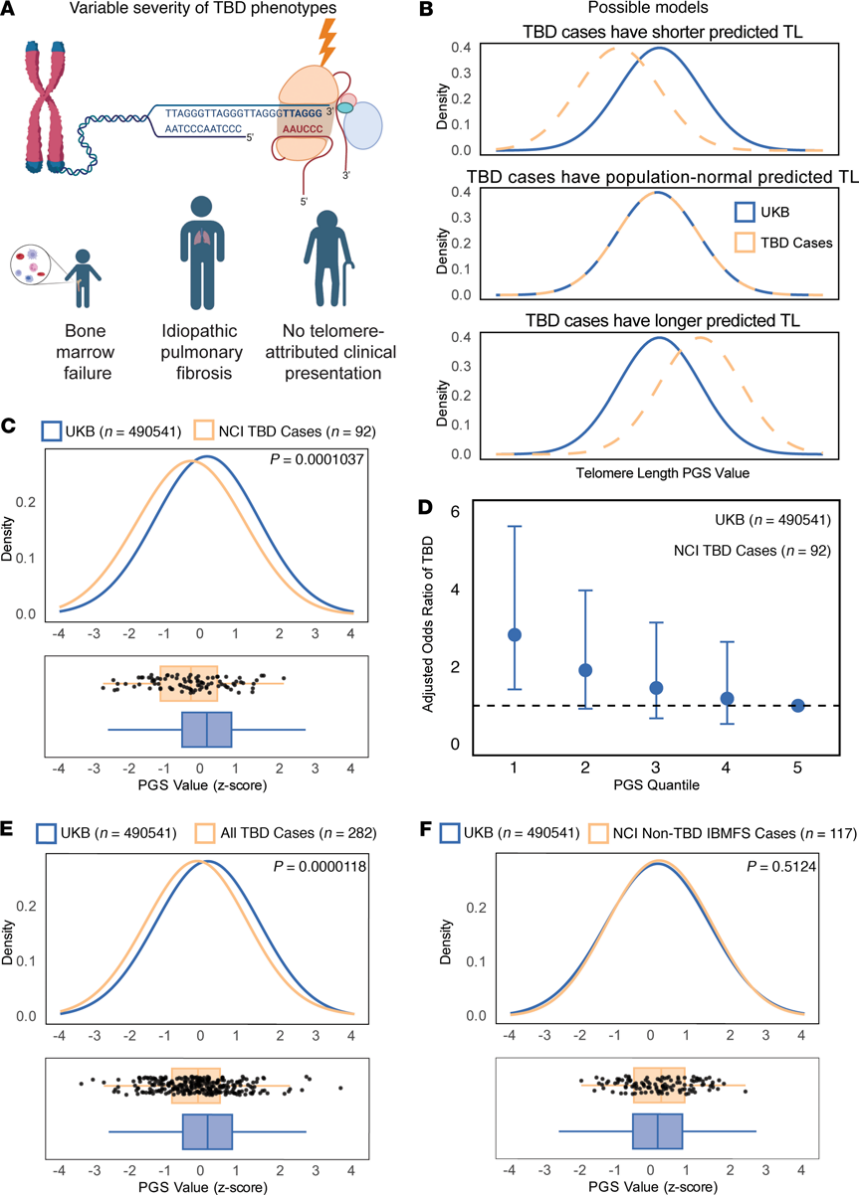
Polygenic modification of TBD expressivity in disease cohorts. (**A**) Illustration. TBD-associated germline variants affect genes involved in telomere length and integrity. Variable expressivity of TBD variants results in diverse phenotypic presentations and age of onset. (**B**) Schematic of distribution of TBD-case telomere length polygenic scores compared with biobanks, under different hypotheses. If common variation affecting telomere length contributes to TBD expressivity and disease cohort ascertainment, left-shifted (towards shorter TL) PGS distribution would be expected (top panel). The null hypothesis is that TBD high-impact variants overpower any effects of common variation (central panel). An alternative hypothesis is that common variation predisposing to long telomere length protects TBD variant carriers from severe phenotypes and mortality; under this model, a right-shifted PGS could be observed (bottom panel). (**C**) Distribution of telomere length PGS in NCI TBD cases compared to the UK Biobank (Welch’s 2-tailed *t* test, *P* = 1.037 × 10^–4^). (**D**) Odds ratio of case-control status versus telomere length PGS quintile, NCI TBD cases. (**E**) Comparison of meta-analysis of telomere length PGS distribution in NCI and DCR cases versus UK Biobank (Welch’s 2-tailed *t* test, *P* = 1.18 × 10^–5^). (**F**) Comparison of NCI non-TBD IBMFS case telomere length PGS versus UK Biobank (Welch’s 2-tailed *t* test, *P* = 0.5124).

We reasoned that if both monogenic germline mutations and polygenic predisposition to short telomeres contribute to the clinical severity of TBDs and the likelihood of early-onset manifestations, the distribution of genetically predicted telomere length in this clinically ascertained cohort would be shifted towards shorter telomeres compared with the population average (Figure 2B). Consistent with this, individuals with TBDs enriched for early-onset bone marrow failure phenotypes (*n* = 92) had a median polygenic score 0.44 SDs shorter than the UK Biobank (*P* = 1.04 × 10^–4^), and 0.37 SDs shorter than the external All of Us cohort (*P* = 5.82 × 10^–4^) (Figure 2C and Supplemental Figure 1A). To estimate the effect size of the polygenic contribution, we binned the PGS distribution into quintiles based on the UK Biobank population distribution and calculated odds ratios with the number of TBD patients in each quintile representing cases and with UK Biobank participants as controls. Individuals in the lowest quintile of genetically predicted telomere length had approximately three-fold odds of being a TBD case compared with those in the highest quintile (Figure 2D). We validated these findings in a separate cohort with 190 TBD patients from the Dyskeratosis Congenita Registry (DCR) at Queen Mary University of London (81). The DCR cohort has a broader referral base and is less enriched for reported severe phenotypes compared with the NCI cohort (80, 81); consistent with this, we observed a slightly attenuated but consistent effect compared with our original NCI discovery cohort (median difference –0.20 SDs, *P* = 0.009) (Supplemental Figure 1B). A combined analysis across both TBD cohorts further demonstrated a consistent and strongly significant association between an individual’s PGS and their odds of having a TBD (median difference –0.28 SDs, *P* = 1.18 × 10^–5^) (Figure 2E and Supplemental Figure 1C).

Interestingly, in both the NCI and DCR cohorts, a subset of patients harbored no known causal high-impact TBD mutation. These patients may have lower effect-size mutations that past TBD gene discovery efforts have been unable to detect. Under a simple liability-threshold model in which rare large-effect variants, common small-effect variants, and environmental effects combine to drive disease risk, patients with no identified large-effect variant are expected to have a more significant polygenic contribution to their disease risk on average (82, 83). To test this, we separated patients with and without a known TBD variant. While both patients with and without a known variant have a significantly shifted PGS predictive of short telomeres compared with the population average, those with no known variant had an increased PGS burden for shorter telomeres compared with individuals with a known variant (Supplemental Figure 1D), though the difference was not statistically significant. While underpowered, this analysis hints at a model in which polygenic variation might contribute to TBD risk and variant penetrance and expressivity.

We considered the possibility that population stratification and other demographic factors contributing to differences in the PGS across populations could underlie our observations (84–87). We used kinship-based inference for GWAS (KING) and 1000 Genomes reference data to infer ancestry in our patient cohorts and the UK Biobank (88, 89). The vast majority of individuals in each of the NCI, DCR, and UK Biobank cohorts were of predominantly European ancestry, followed by Admixed American ancestry (Supplemental Figure 1E, Supplemental Table 18). In conducting the GWAS and constructing the polygenic scores, we restricted our analyses to the European ancestry subset in the UK Biobank to minimize the effects of population stratification and maximize predictive accuracy in the TBD patients (see Methods). We closely examined our results to determine if ancestry had a significant effect. We found that the PGS distribution did not differ between European and non-European individuals in the patient cohorts (*P* = 0.87) (Supplemental Figure 1F). Furthermore, the PGS in the cohorts was not associated with the ancestry principal components inferred by KING (Supplemental Figure 1G) (84–87). As a negative control, we also assessed cases of non-TBD inherited bone marrow failure syndrome cases that included individuals diagnosed with Diamond-Blackfan Anemia, Fanconi Anemia, and Shwachman-Diamond Syndrome, and that came from the same NCI bone marrow failure syndrome cohort as the patients with TBD. For these conditions, telomere length does not drive the disease process, and nontelomere related gene mutations are implicated (71, 72, 90–92). As expected, the polygenically predicted telomere length for these patients is indistinguishable from the UK Biobank population average (*P* = 0.51) (Figure 2F), and the telomere length PGS for the TBD patients was 0.49 SDs shifted towards shorter predicted telomeres compared with non-TBD–inherited bone marrow failure syndrome patients (*P* = 3.99 × 10^–4^), indicating that the observed phenomenon is telomere disease-specific and is unlikely to be due to population stratification (Supplemental Figure 1H).

### Polygenic variation associated with telomere length impacts TBD penetrance and expressivity in population biobanks.

Taken together, our analyses of the NCI and DCR cohorts indicate that common genetic variants associated with short telomere length contribute to ascertainment as a TBD case in disease cohorts enriched for individuals with childhood-onset bone marrow failure. We reasoned that the reverse should also be true: TBD causal variants should be present in adult population biobanks, and adults with a pathogenic variant who avoided the severe childhood-onset manifestations of TBDs should not have polygenically predicted short telomere length (Figure 2A). To test this, we examined the UK Biobank for carriers of variants in the genes known to cause TBDs (see Methods and Supplemental Table 3).

To be maximally comprehensive while maintaining stringency, we defined multiple variant sets, given that each variant annotation approach has limitations for predicting true pathogenicity (93). First, we included variants annotated in ClinVar as causing dyskeratosis congenita or a related TBD with high confidence, hereafter referred to as “ClinVar Pathogenic.” Applying quality-control, dominance, and ancestry filters, we identified 213 variant carriers (Figure 1B). Next, we defined a more restrictive subset of ClinVar variants including only carriers of variants specifically annotated to cause childhood-onset TBDs in a dominant manner and male carriers of *DKC1*, resulting in 22 variant carriers, referred to as “ClinVar Dominant-Acting.” Finally, to be maximally inclusive of potentially pathogenic variants that may not have been annotated in ClinVar, we defined a set of predicted pathogenic rare coding variants in TBD genes using a consensus of Ensembl Variant Effect Predictor, LOFTEE, and AlphaMissense annotations, resulting in 1,666 carriers in the UK Biobank (“Consensus Predicted Pathogenic”) (see Methods and Supplemental Tables 4–12) (94–96). Genes that harbor TBD-causal mutations are often classified based on mode of inheritance (16, 17, 97). For all of these analyses, we excluded genes that cause TBDs in an exclusively autosomal recessive manner, given the challenges present in determining the phase of mutations (see Methods).

**Figure 3:**
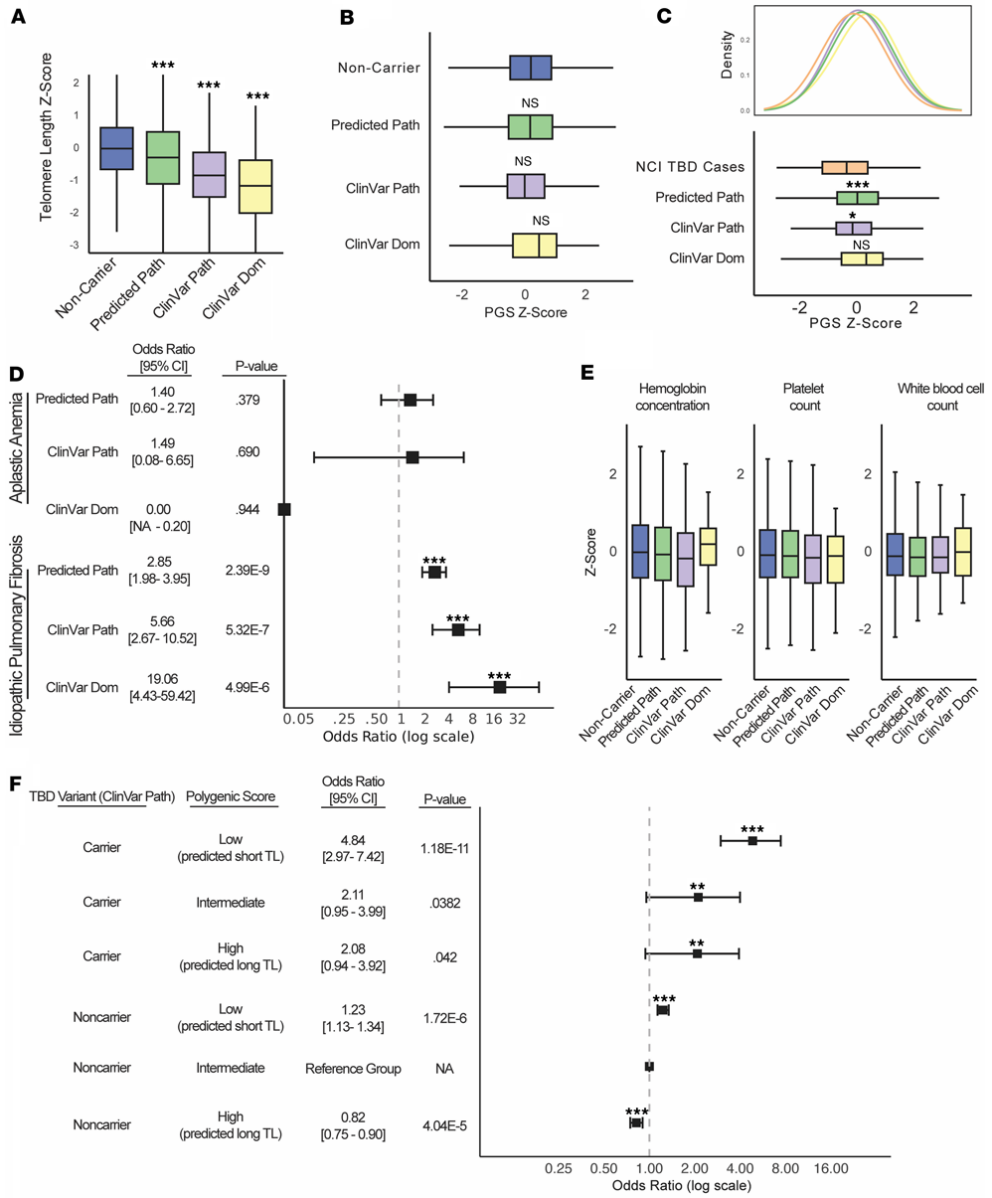
Polygenic modification of TBD expressivity in the UK Biobank. (**A**) Measured telomere length in UK Biobank noncarriers and carriers of pathogenic TBD variants (pairwise 1-sided *t* tests with Bonferroni multiple testing correction; noncarrier versus Predicted Pathogenic: *P* = 3.80 × 10^–20^; noncarrier versus ClinVar Pathogenic: *P* = 3.46 × 10^–23^; noncarrier versus ClinVar Dominant-Acting: *P* = 1.39E-3). (**B**) TL PGS in UK Biobank noncarriers and carriers of pathogenic TBD variants (pairwise 2-sided *t* test with Bonferroni multiple testing correction; non-carrier versus Predicted Pathogenic: *P* = 1; noncarrier versus ClinVar Pathogenic: *P* = 0.20; noncarrier versus ClinVar Dominant-Acting: *P* = 1). (**C**) TL PGS in NCI cases compared to UKB pathogenic variant carriers (pairwise 1-sided *t* test with Bonferroni multiple testing correction; TBD case versus Predicted Pathogenic: *P* = 0.00087; TBD case versus ClinVar Pathogenic: *P* = 0.041; TBD case versus ClinVar Dominant-Acting: *P* = 0.0896). (**D**) Odds ratios of aplastic anemia and idiopathic pulmonary fibrosis in carriers of pathogenic TBD variants compared with noncarriers (logistic regression adjusting for age and sex). (**E**) Blood cell counts in UK Biobank noncarriers and carriers of pathogenic TBD variants (pairwise *t* test with Bonferroni multiple testing correction). (**F**) Odds ratios of idiopathic pulmonary fibrosis in UK Biobank stratified by PGS tertile (top third, middle third, and lowest third) and ClinVar Path or Predicted Path variant-carrier status, using noncarrier intermediate group as the control group (logistic regression adjusting for age, sex and first 4 ancestry PCs).

Supporting the pathogenicity of the variants in the associated sets, individuals in each group in the UK Biobank had shorter measured telomere length (TL) compared with noncarriers. Furthermore, the magnitude of effect was concordant with the expected order of pathogenicity, with the most inclusive Consensus Predicted Pathogenic cohort associated with the smallest average decrease in TL (0.31 SDs), the more restrictive ClinVar Pathogenic cohort associated with a 0.85 SD decrease in mean TL, and the most restrictive ClinVar Dominant-Acting cohort showing the largest average decrease in TL (1.15 SDs) (Figure 3A).

Despite having short telomeres, all three variant carrier cohorts had a population-normal polygenic contribution to telomere length on average compared with noncarriers of TBD variants in the UK Biobank (Figure 3B), and significantly longer predicted telomere length than the TBD cohorts (Figure 3C). Interestingly, the 22 carriers of the most severe mutations (“ClinVar Dominant-Acting”) appeared to have a right-shifted polygenic score distribution relative to noncarriers, suggestive of a protective effect that could help explain why carriers of these large-effect variants escaped early-life manifestations of disease, but this difference was not statistically significant given the small sample size (Figure 3B). These findings complement our results in patient cohorts, demonstrating that individuals with TBD mutations who do not have early clinical presentations have a relatively decreased common variant burden for short telomeres compared with childhood-onset disease cohorts. Together, these findings support the idea that the risk of childhood-onset severe TBD manifestations due to large-effect causal TBD gene variants can be modified by common variants that impact telomere length.

We then assessed whether these pathogenic variant carriers were enriched for childhood or adult-onset TBD phenotypes relative to noncarriers in the UK Biobank. Importantly, examining childhood-onset TBD phenotypes, we found no evidence for increased risk of bone marrow failure or altered blood counts in these TBD variant carriers (Figure 3, D and E). We then examined idiopathic pulmonary fibrosis, an adult TBD manifestation (97, 98). We found that being a carrier of a pathogenic variant was associated with greatly increased odds of presenting with idiopathic pulmonary fibrosis (Figure 3D). We wondered whether common variation also affects the penetrance of these adult-onset manifestations of TBDs. Strikingly, we found that within both variant carriers and noncarriers, telomere length PGS stratified risk of idiopathic pulmonary fibrosis (Figure 3F and Supplemental Figure 2D, combined VEP and ClinVar carrier analysis and separated, respectively).

We sought to quantify the effects of PGS in both rare TBD variant carriers and noncarriers and also asked whether there was evidence for a nonadditive interaction between polygenic effects on telomere length and pathogenic variants. We regressed idiopathic pulmonary fibrosis disease status on PGS, variant carrier status, and an interaction term between the two, while controlling for age, sex, and the first 4 ancestry principal components (Supplemental Table 13). Including both carriers and noncarriers, a 1 SD decrease in PGS (predicting shorter telomere length) was associated with an odds ratio of 1.22 for idiopathic pulmonary fibrosis (*P* = 9.04 × 10^–27^). The included interaction term between rare variant status and PGS was not statistically significant and did not affect the coefficient estimates (Supplemental Table 14). Restricting to only rare variant carriers, this association was consistent, but not statistically significant (though possibly limited in statistical power due to small sample size), with a 1-unit decrease in PGS associated with an odds ratio of 1.31 for having idiopathic pulmonary fibrosis (*P* = 0.108) (Supplemental Table 15). We confirmed through a mediation analysis that the effect of telomere length PGS on pulmonary fibrosis risk is mediated through telomere length (Supplemental Table 16 and Supplemental Note) (99). These results support a model in which small-effect polygenic variants and pathogenic large-effect variants independently contribute to adult TBD manifestations by affecting telomere length and maintenance, although this analysis may be limited by power (see Methods).

In summary, we found that carriers of TBD-causing variants in the UK Biobank, in contrast to TBD cohorts, have a population-normal PGS and no enrichment for bone marrow failure, but do have increased risk of idiopathic pulmonary fibrosis, a common adult manifestation of TBDs. While the UK Biobank variant carriers were not enriched for PGS associated with short telomere length overall, common genetic influences captured by the PGS do impact the penetrance of TBD mutations associated with idiopathic pulmonary fibrosis. Collectively, these findings demonstrate that both pathogenic mutations and common genetic variation associated with telomere length combine to impact penetrance and expressivity.

### Within-family polygenic effects on disease risk.

**Figure 4:**
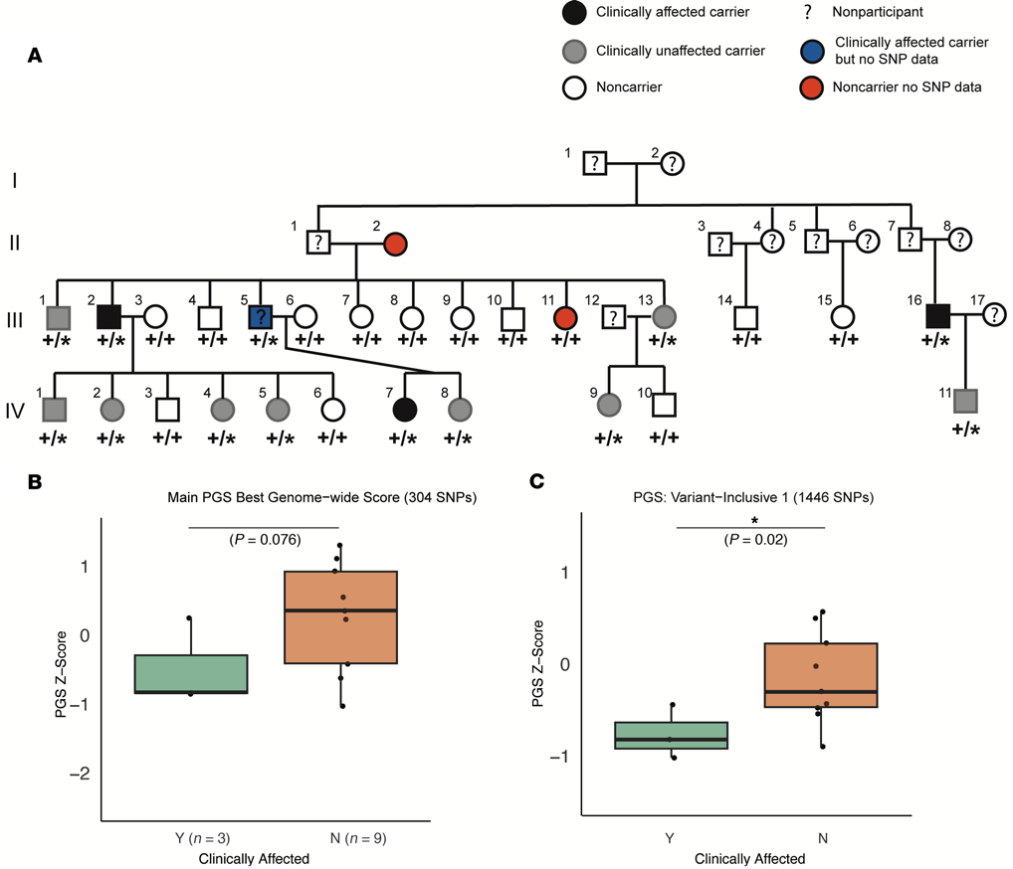
Polygenic modification of expressivity within a family. (**A**) Pedigree depicting family with TERT variant. Black, case; gray, TERT noncase carrier; transparent, noncarrier; square, male; circle, female; ?, unknown status; diamond, unknown sex. (**B**) Telomere length PGS comparing cases with noncase TERT variant carriers using the same parameters as best genome-wide SNP score (linear mixed model with kinship matrix as random effect; see Methods). (**C**) Telomere length PGS comparing cases with noncase TERT variant carriers for Variant-inclusive PGS Score 1 (linear mixed model with kinship as random effect).

Having observed a significant effect of common polygenic variation associated with telomere length on clinical manifestations of TBDs in large disease cohorts and population biobanks, we wondered whether polygenic variation also affects penetrance and expressivity within a single family with a shared causal variant. To explore this question, we analyzed a large kindred with multiple dyskeratosis congenita cases and a heterozygous pathogenic variant in the *TERT* gene (ClinicalTrials.gov Identifier: NCT00027274). Of the 22 family members for whom genotype data was available, there were 12 carriers of the pathogenic *TERT* variant, 3 of whom had clinically diagnosed TBDs (Figure 4A). We restricted all analyses to the 12 *TERT* mutation carriers, comparing the 3 clinically affected family members with the 9 unaffected family members.

We reasoned that the strict clumping and pruning approach that was optimal to construct polygenic scores in the UK Biobank may not be best suited to detect relatively subtle within-family common genetic variation, given significant shared variation among the family members. Therefore, alongside the PGS used throughout the study, we constructed multiple other polygenic predictors, including more variants, reasoning that this would be more likely to pick up any subtle differences that exist within a family. As an orthogonal approach, we also constructed a polygenic score including all conditionally genome-wide significant SNP signals (100), with the idea that this would enable detection of multiple independent effects on different haplotypes that could be segregating within this family (100). We accounted for family structure using a linear mixed model with kinship as a random effect. Remarkably, across all tested approaches, the clinically affected family members had a more negative PGS on average than the unaffected variant-carrying family members, indicating a greater burden of polygenic variation associated with shorter telomeres (Figures 4, B and C, and Supplemental Figure 3, B and C). We speculate that the affected family members have disease not because they have a shifted PGS causing a somewhat shorter mean telomere length, but rather that the PGS hints that these patients present with disease at least in part because of a decreased general ability to protect and repair telomeres, on the background of a major perturbation caused by the rare disease-driving mutation.

Thus, even in the relatively controlled setting of a family with a shared causal variant and overall similar genetic background, our results suggest that random segregation of common variants that alter disease biology might contribute to variable expressivity and penetrance. These results provide a framework for larger studies of trios and families to further elucidate how common genetic variation may help explain why some family members with a disease-causing variant have severe symptoms, while others remain clinically unaffected.

### Convergence of common and rare genetic variation in telomere biology disorders.

A key question regarding polygenic modifiers of disease is whether common and rare variation converges on the same genes and biological pathways (101). In autism spectrum disorder, examples of convergence of common and rare variation at the same loci have been observed (102). Similarly, in Hirschsprung’s disease and craniosynostosis, shared signaling and regulatory pathways are affected by rare and common risk variants (6, 9, 12). In contrast, in sickle cell disease and β-thalassemia, common genetic variation largely impacts disease expressivity through modulation of fetal hemoglobin gene transcriptional regulation, a mechanism distinct from the primary disease-causing mutations that alter adult hemoglobin (10, 103). Having shown that common polygenic variation affects penetrance and expressivity in TBD, we asked whether these variants act upon the same or different genes as the causal high-impact variants underlying TBDs.

**Figure 5:**
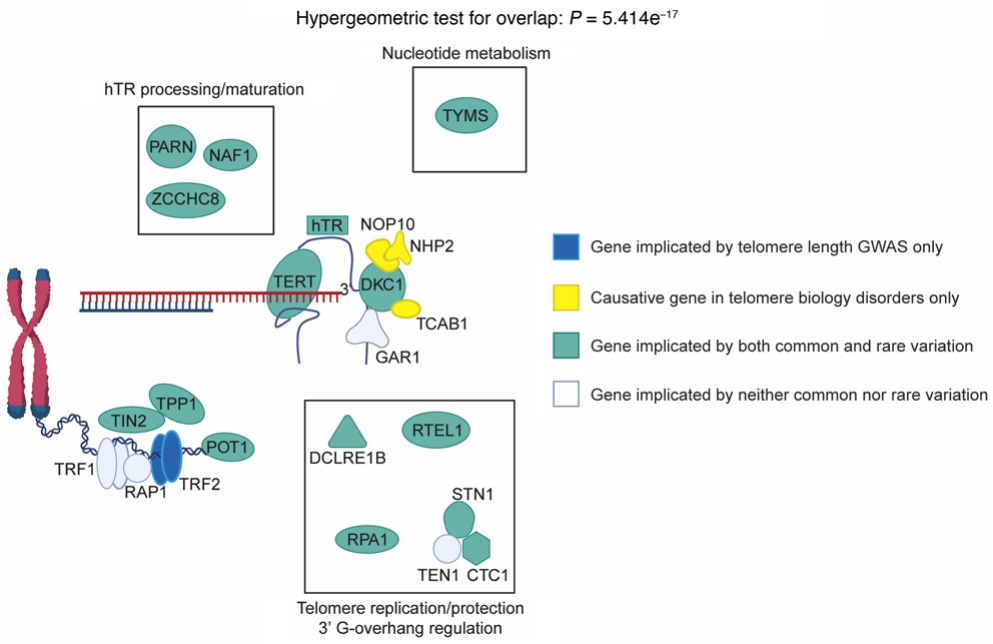
Common and rare genetic variation converges in telomere biology disorders. Genes with mutations that cause TBDs show strong overlap with genes underpinning common-variant associations with telomere length (hypergeometric test). Causal genes associated with common SNPs in telomere length PGS overlaid with genes causally implicated in telomere biology disorders.

In the TBDs, causal variants affect genes regulating telomere length, maintenance, and function (17, 97). Using multiple gene prioritization approaches (see Methods), we found that common variation associated with telomere length and TBD expressivity implicates genes that strongly overlap the set of genes implicated as high-impact monogenic variants in TBDs (*P* = 5.41×10^–17^) (Figure 5 and Supplemental Figure 4A). The polygenic variants are primarily noncoding, with more than 97% of credible set variants mapping to introns or intergenic regions (Supplemental Figure 4B). These variants show a striking enrichment for enhancers in CD34^+^ hematopoietic stem and progenitor cells, possibly explaining the association we observe in bone marrow failure and likely underlying mechanisms in these progenitors for all blood and immune cells (Supplemental Figure 4C). Collectively, these findings suggest a model in which common, noncoding variation converges upon the same genes implicated by high-effect Mendelian coding mutations.

## Discussion

Variable expressivity and incomplete penetrance are important challenges for rare, monogenic disorders (1). Because telomere length is a measurable population-level trait, the TBDs provide a unique opportunity to test the contribution of common genome-wide variation to phenotype expressivity in a rare, presumed monogenic disease. By utilizing both disease cohorts enriched for severe, childhood-onset TBD phenotypes as well as population biobanks that are depleted of individuals with severe pediatric disease (104), we show that even in a rare-disease setting, where pathogenic variants are expected to be highly penetrant, common variation plays an important role in determining phenotype expression.

First, we show that common variation affecting telomere length influences the severity and clinical presentation of telomere biology disorders. We find that individuals in disease cohorts enriched for cases with severe childhood-onset TBD manifestations have a left-shifted PGS compared to the general population, indicating that polygenic variants associated with telomere length contribute to disease liability. Importantly, there is phenotypic heterogeneity in these cohorts, and we might underestimate the impact of polygenic variation as a result. Next, we show that carriers of these same variants in the UK Biobank, a population depleted of severe childhood disease, have a population-normal PGS. Consistent with this, variant carriers in the UK Biobank do not have enrichment for severe childhood-onset TBD manifestations, but are enriched for the adult-onset TBD presentation of idiopathic pulmonary fibrosis. We also show that telomere length PGSs and rare variants independently contribute to the likelihood of having idiopathic pulmonary fibrosis in the UK Biobank. We further suggest that, within a single family sharing a rare disease-causing variant, common genetic variation might affect an individual’s likelihood of developing the disease. Finally, we show that common, noncoding variants and rare, highly penetrant coding variants converge on genes that regulate telomere length and maintain telomere homeostasis.

Our work builds upon previous studies that have shown that polygenic variation interacts with high-impact variants in relatively more common disease settings (3, 4, 7, 8). Here, we have identified an example of polygenic modifiers impacting an extremely rare disease by affecting the same genes and biological mechanisms as high-impact rare variants. Future work will likely uncover other examples, as exemplified through studies of disease modifiers such as fetal hemoglobin, where modifiers affect phenotypes through distinct biological mechanisms (10, 103). Increasingly large population sequencing studies have identified many individual rare and common variant associations with traits (93). However, for many phenotypes, it is still challenging to explain why some variant carriers will present with severe disease, while others will have more moderate presentations, or not have any clinical manifestations at all (1). Our findings lay the groundwork for future work to precisely quantify the polygenic contribution to penetrance and expressivity across a range of common and rare diseases. For the TBDs specifically, penetrance and expressivity have been discussed as important clinical challenges, but most prior work has focused on the importance of high-impact rare coding variants (17, 97). Our work establishes common variation as a contributor to this phenomenon and lays the groundwork for detailed clinical studies delineating the prognostic and therapeutic value of common modifiers of telomere length for TBDs.

### Limitations.

Our analyses are based on average telomere length in white blood cells, which is only a correlated proxy for the length of the shortest telomere, which is likely the more biologically meaningful measurement in telomere homeostasis and the TBDs (105–107). Furthermore, telomere length is correlated yet variable across cell and tissue types, and TBDs are a multisystem disease. While mean leukocyte TL is the best available metric to assess common genome-wide variation impacting telomere homeostasis, it provides an imperfect estimate of the common genetic variant effects that contribute to penetrance and expressivity in the TBDs, and better telomere measurements across multiple tissues will enable more precise future population-level analyses (108, 109). Moreover, both disease cohorts and population biobanks have strengths and weaknesses for assessing variant penetrance and expressivity. Disease cohorts may overestimate variant pathogenicity, and adult biobanks are likely to underestimate variant impact (110). Different adult biobanks have variable ascertainment characteristics, which can affect estimates of variant expressivity (104, 110–112). For this reason, in this study, we utilize both disease cohorts and include both the UK Biobank and All of Us cohorts, which have different recruitment mechanisms and demographic characteristics, enabling complementary insights.

Finally, TBDs are heterogeneous; mode of inheritance and effects of specific causal variants have been shown to be associated with variation in outcome (16). In this study, we grouped different genotypes together to power analyses of the effects of common genetic variation, likely obscuring some effect heterogeneity. Other factors may contribute to variable expressivity, including genetic anticipation, environmental influences, and somatic genetic rescue (17). Future, larger studies will be necessary to tease out the effects of variant- and gene-specific interactions and other mechanisms underlying variable expressivity and incomplete penetrance.

## Methods

### Sex as a biological variable

For all analyses, individuals of both male and female sex were included (Supplemental Table 17). For genome-wide association studies and PGS construction, sex was included as a covariate. For statistical testing, sex was included as a covariate to assess the significance of biological sex for effects studied, as detailed in the relevant Methods sections below.

### Study participant details

#### UK Biobank.

The UK Biobank is a large prospective cohort with extensive phenotype and molecular data available, including whole-genome sequencing (113, 114). The UK Biobank was accessed under application number 31063. Cohort analysis details are included here under Method Details – *UK Biobank*.

#### All of Us.

All of Us is a longitudinal cohort that currently contains short-read whole genome sequencing data and phenotypic data from 245,394 participants. Whole genome sequencing was performed on participants to a mean depth of 30 ×. Informed consent for all participants is conducted in person or through an eConsent platform that includes primary consent, HIPAA Authorization for Research use of EHRs and other external health data, and Consent for Return of Genomic Results. The protocol was reviewed by the Institutional Review Board (IRB) of the All of Us Research Program. The All of Us IRB follows the regulations and guidance of the NIH Office for Human Research Protections for all studies, ensuring that the rights and welfare of research participants are overseen and protected uniformly. Cohort analysis details are under Method Details – *All of Us*.

#### National Cancer Institute inherited bone marrow failure syndrome samples.

Processed genotype array files were obtained as part of the NCI Inherited Bone Marrow Failure Study (ClinicalTrials.gov Identifier: NCT00027274). Genotype data was available for 92 dyskeratosis congenita cases, 49 Diamond-Blackfan Anemia cases, 45 Fanconi Anemia cases, and 19 Shwachman-Diamond Syndrome cases. Genotype data was also available for 22 members of a family with a shared *TERT* mutation that included 4 dyskeratosis congenita cases. Genotype data was also available for 120 unaffected family members of dyskeratosis congenita cases in the cohort. Deleterious variants were originally identified in these patients using TBD gene sequencing panels.

#### Dyskeratosis Congenita Registry Queen Mary University of London samples.

Two sample sources were used as part of the DCR cohort. The first included 49 samples of unknown mutation that had previously undergone sequencing. For the second, DNA from 132 cases of known genotype was sequenced using low-pass whole-genome sequencing (mean coverage 0.5 ×) by Azenta Life Sciences.

See Supplemental Methods for processing details for each cohort.

### Method details — Genome-wide association analysis for telomere length

#### Phenotype and sample construction.

For the telomere phenotype, we used the qPCR-based leukocyte telomere length measurements generated, batch-corrected, and quality controlled in 474,074 UKB participants (UKB Data Field 22191) (74). The phenotype was log-normalized and z-scored as in Codd et al. (73). Of the remaining individuals, we then removed the individuals with the highest and lowest 0.5% of telomere length values to minimize the impact of outliers (Supplemental Table 19).

The set of filtered, phenotyped individuals was randomly split into 2 nonoverlapping subsets. Two-thirds of the individuals (*n* = 259,142) were used in the GWAS analysis of telomere length, and the remaining one-third of samples (*n* = 129,547) were used for subsequent polygenic risk score construction.

#### Genome-wide association study for telomere length.

GWAS was performed with genotype array data for REGENIE Step 1 and whole-genome sequencing data for REGENIE Step 2. Age, sex, and the first 10 genetic principal components were included as covariates. Additional QC, filtering parameters, and full details are provided in Supplemental Methods – *UK Biobank*.

### Method details — Polygenic risk score construction using telomere length summary statistics

#### Primary polygenic risk score construction.

The summary statistics and processed whole-genome sequence variant call files were then used to construct polygenic risk scores using the 129,547 samples that were not included in the GWAS. As the goal of constructing these scores was for use as an instrument to assess the burden of common genetic variation affecting telomere length across different cohorts, we restricted analysis to SNPs that were well imputed (INFO > 0.6) across each of the different cohorts that were tested. This retained 5,279,945 SNPs across the genome.

Polygenic scores were constructed using the common clumping and thresholding approach. In clumping and thresholding, index SNPs are selected, and SNPs within a prespecified distance window and above a prespecified correlation threshold are identified (“clumped”) and removed (“pruned”), to remove redundant effects from SNPs on the same haplotype, and this process is iteratively repeated across the genome. For the variants remaining after pruning, a range of *P*- value thresholds is then applied, removing all variants above the *P*-value threshold. The resulting variant sets are then used to compute polygenic scores defined as the sum of variant allele counts weighted by effect size estimates from the telomere length GWAS. See Supplemental Methods for details on score selection, which yielded the best performing polygenic score with 304 genome-wide SNPs, and a variance explained for telomere length of 0.071 (Supplemental Table 1).

#### Comparing polygenic risk scores across cohorts.

The SNPs that were included in the best PGS score computed in the UK biobank were used to directly compute scores in each different cohort. See Supplemental Methods – *Primary polygenic risk score construction* for details.

#### All of Us polygenic risk score construction and testing.

To compare telomere length PGS between the All of Us (111) cohort and patient samples, an identical construction procedure was followed as in UK Biobank, but restricting to SNPs genotyped with high quality in All of Us. See Supplemental Methods – *All of Us* for details.

#### Plotting and comparing score distributions.

See Supplemental Methods – *Plotting and comparing score distributions* for details.

#### Computing odds ratios.

To assess the relationship between PGS and the outcome of dyskeratosis congenita, we defined individuals with dyskeratosis congenita as cases and UK Biobank participants as controls. The PGS population values were binned into 5 equal-sized quintiles, and each observation for both cases and controls was placed in the appropriate quintile. We then calculated odds ratios and 95% confidence intervals for each quintile relative to the lowest risk (longest predicted telomere length) quintile. For each quintile, a 2 × 2 contingency table was constructed, comparing the number of outcome cases within the quintile to those outside it. Fisher’s exact test was applied to estimate the odds ratio for each quintile. The standard error of the logarithm of the odds ratio was used to compute the 95% confidence intervals.

#### Ancestry inference.

See Supplemental Methods – *Ancestry inference* for details.

### Method details — UK Biobank pathogenic variant carrier analysis

#### Defining variant sets.

For all analysis of variant carriers in the UK Biobank, the following genes were used as a TBD gene set, defined based on genes with strong evidence of being capable of causing dyskeratosis congenita, Revesz syndrome, Hoyeraal-Hreidarsson syndrome, or Coats plus syndrome, and having a mode of inheritance reported in the literature as causing dyskeratosis congenita/other syndromic TBDs, or bone marrow failure in a monoallelic or “monoallelic or biallelic” fashion, excluding genes for which only biallelic inheritance has been reported (Supplemental Table 2) (17). This resulted in inclusion of the following genes: *TERC*, *TINF2*, *ZCCHC8*, *ACD*, *RTEL1*, *TERT*, and *DKC1*. For *DKC1*, only male carriers (X-linked recessive) were included. Noncarriers were defined as any participants passing QC that did not carry one of the pathogenic variants included in the final analyses. See Supplemental Methods *– Defining variant sets* for details of the ClinVar query.

The Predicted Pathogenic dataset was produced starting from all variants in the whole exome dataset that passed QC (above) with less than 1% frequency, within the start and end positions of known TBD genes (NCBI). Variants were then annotated using the Ensembl Variant Effect Predictor. The annotations were then filtered to retain all predicted “HIGH IMPACT” variants. Variants with predicted “MODERATE IMPACT” were further annotated using the AlphaMissense released pathogenicity scores, and variants predicted as “likely_pathogenic” were retained (94, 95).

#### Pre-processing the UK Biobank whole-exome sequencing data.

See Supplemental Methods.

#### Identification and phenotypic analysis of carriers of whole-exome variants.

We used the UK Biobank Research Analysis Platform to extract phenotype data. We first filtered individuals using the criteria described in Supplemental Methods – *Pre-processing the UK Biobank whole-exome sequencing data* for quality control and White British ancestry. We extracted phenotypic information on blood cell counts, clinical records, and telomere length. To assess for blood cell count differences, the Haemoglobin_concentration, Platelet_count, and White_blood_cell_count phenotype fields were utilized and z-scored. To assess for idiopathic pulmonary fibrosis, we used ICD-10 code of J84.1 (“other interstitial pulmonary diseases with fibrosis”). To assess for aplastic anemia, we used ICD-10 code of D61 (“Other aplastic anaemias,” which includes “Aplastic anaemia, unspecified,” “constitutional aplastic anaemia,” “idiopathic aplastic anaemia,” “other specified aplastic anaemias,” and “aplastic anaemia, unspecified”).

#### Statistical analyses of UKB pathogenic variant carriers.

See Supplemental Methods – *Statistical analyses of UKB pathogenic variant carriers* for details on statistical analyses, interactions between PGS and rare variant status, and mediation analysis.

### Method details — Analysis of expressivity within a family

#### Pedigree analysis polygenic risk score construction.

We utilized the same set of 5,279,945 SNPs to compute polygenic risk scores in the pedigree cohort. We constructed 4 different polygenic scores. Three were constructed taking the best score using clumping and thresholding with various sets of hyperparameters, while one score was constructed using only COJO genome-wide conditionally independent SNPs, as an orthogonal approach. See Supplemental Methods – *Pedigree analysis polygenic risk score construction* for details. Each pedigree score was validated by binning the PGS into four quartiles and computing the Spearman correlation between the quartile and measured telomere length in the UK Biobank.

#### Family data statistical analysis.

Because of high relatedness within the pedigree, a linear mixed model with a kinship matrix as a random effect was used. The kinship matrix was generated using PLINK (-- make-rel square) using the pedigree genotypes to estimate relatedness. The LMM was performed using lmm.aireml, with the PGS and an intercept term as fixed effects and the kinship matrix as a random effect. Fixed effects estimates and standard errors were extracted from the LMM results. Wald test statistics were computed as the ratio of the fixed effects to their standard errors, and *P* values were derived from the Wald test statistics.

### Method details — Analysis of convergence of common and rare variation

#### Gene prioritization.

From the telomere length GWAS summary statistics, genes were prioritized using MAGMA and a closest-gene approach based on COJO conditionally genome-wide significant SNPs. See Methods – *Gene prioritization* for details.

#### Finemapping.

See Supplemental Methods for details.

#### Fine-mapped variant annotation.

Fine-mapped variants were annotated using the Ensembl VEP and the Activity-by-Contact genome-wide enhancer maps (94, 115). A credible set of fine-mapped variants was defined as variants with posterior inclusion probability greater than 0.9. This resulted in a fine-mapped set of 529 variants. Credible set variants were annotated using Ensembl VEP for functional sequence type. ABC enhancer maps from CD34^+^ hematopoietic stem cells were overlapped with the credible set variants. Enrichment of fine-mapped variants within CD34^+^ enhancers was assessed by comparing the proportion of credible set variants within the ABC CD34^+^ enhancers to the proportion of variants with a posterior inclusion probability less than 0.1 within the ABC CD34^+^ enhancers, using a χ^2^ test.

### Statistics

Statistical tests used for each analysis can be found in the figure legends with further details and explanations for choice of test in the relevant METHOD DETAILS sections. These statistical tests were employed: pairwise Welch’s *t* tests with Bonferroni multiple testing correction for comparisons between noncarriers and pathogenic carrier sets as well as for measured telomere length and PGS distributions; Welch’s *t* test for comparing PGS distributions between cohorts (NCI TBD cases versus UK Biobank; NCI TBD cases versus All of Us); logistic regression to estimate odds ratios between pathogenic variant carrier status and PGS tertiles and disease outcomes (IPF, aplastic anemia), adjusting for age, sex, and ancestry PCs; mediation analysis via the R mediator package to quantify the indirect effect of PGS on IPF through telomere length; a linear mixed model with kinship matrix as a random effect and Wald statistics for within-family PGS analyses; Spearman correlation between PGS quartile and adjusted telomere length to validate pedigree PGS scores; Chi-squared tests for enrichment of fine-mapped variants within ABC enhancers; and hypergeometric tests to assess enrichment of known TBD causal genes among MAGMA/closest-gene–prioritized sets. All multiple-comparison analyses were Bonferroni-corrected with significance set at adjusted *P* < 0.05. All statistical tests were performed in R.

### Study approval

All uses of patient data were approved by the Institutional Review Boards at Queen Mary University, the NCI, Boston Children’s Hospital, and the Broad Institute. Written informed consent for use of all patient data was obtained.

### Data availability

Per biobank policy, UK Biobank individual-level data are available upon request by application (https://www.ukbiobank.ac.uk/). TOPMed individual-level genomic data are available by application. Due to patient confidentiality, deidentified patient cohort data will be made available in restricted fashion upon reasonable request to the corresponding author. The data underlying the analysis of finemapped variants in Figure 5 and Supplemental Figure 4 are available in the Supporting Data Values spreadsheet. Due to limitations on sharing individual-level data, summary data information for most other analyses are available in the Supporting Data Values spreadsheet. Further information is available in Supplemental Methods.

## Author contributions

MP and VGS conceived and designed the study. MP and UPA performed computational and statistical analyses. AW, LJM, NG, LPZ, HT, and SAS provided genotype and phenotype data from disease cohorts. AW, LJM, MRN, NG, AG, MJM, HT, and SAS contributed ideas and insights on the analyses. MP and VGS wrote the manuscript with input from all authors. VGS supervised all analytical aspects of this work.

## Supplementary Material

Supplemental data

ICMJE disclosure forms

Supplemental tables 2 and 6

Supporting data values

## Version 1 06/03/2025

In-Press Preview

## Version 2 08/15/2025

Electronic publication
