# Supplementary material for: Polygenic modifiers impact penetrance and expressivity in telomere biology disorders
Source: J Clin Invest. 2025 Jun 3;135(16):e191107. doi: 10.1172/JCI191107 (PMC12352891; doi:10.1172/JCI191107)
Supplement: Supplemental data [file jci-135-191107-s006.pdf]

Supplemental Figures

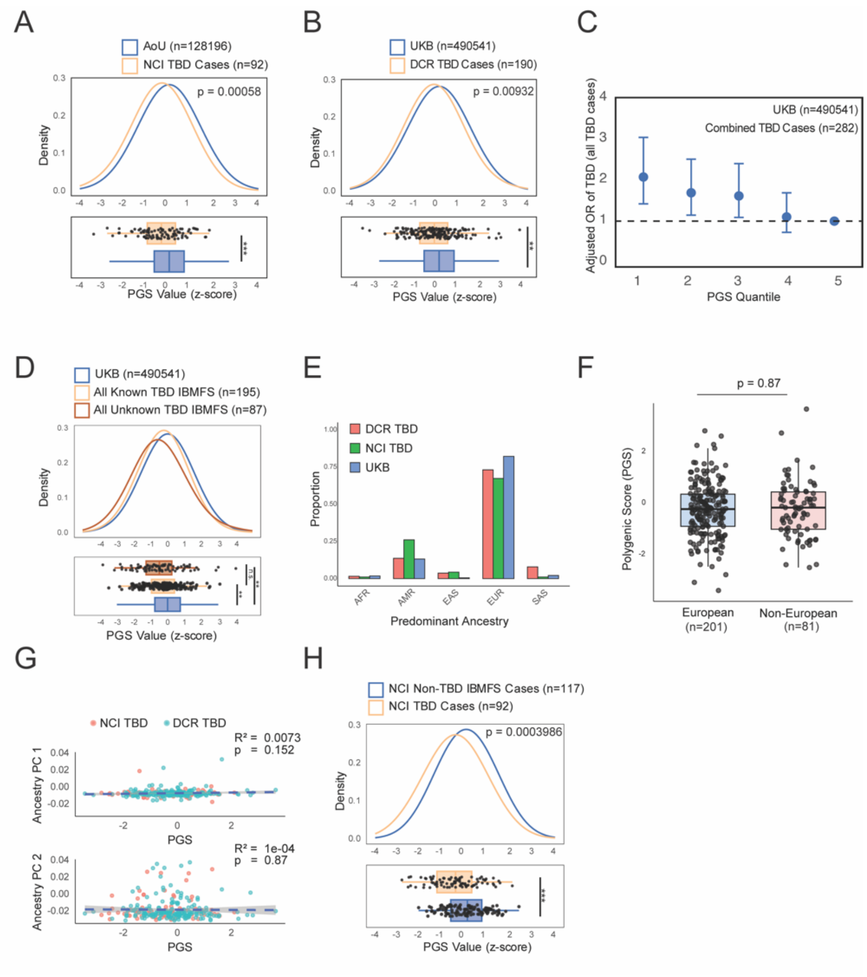

Supplemental Figure S1: Analysis of TBD patient cohorts

A) Distribution of telomere length PGS in NCI TBD cases compared to All of Us cohort (Welch's two-tailed t-test, p = 5.8E-4).

B) Distribution of telomere length PGS in DCR cases compared to the UK Biobank (Welch's two-tailed t-test, p = 9.32E-3).

C) Odds ratio of case-control status versus telomere length PGS quintile, meta-analysis of NCI and DCR cases vs UK Biobank.

D) Comparison of all meta-analyzed cases with known or unknown TBD-causing mutations to UK Biobank (pairwise Welch's two-tailed t-test: UKB vs known IBMFS cases: p = 1.393E-3; UKB vs unknown IBMFS cases: p = 2.788E-3; known vs unknown IBMFS cases: p = 0.2157).

E) Inferred ancestry for DCR and NCI cohorts and UKB participants.

F) Comparison of PGS distribution between European and non-European ancestry DCR and NCI TBD cases (Welch's two-tailed t-test, p = 0.87).

G) Association between ancestry PCs and polygenic score distribution (displayed are Pearson R^2 values and p-values from linear regression of PGS regressed on PC1 or PC2).

H) Comparison of telomere length PGS between NCI TBD cases and NCI non-TBD IBMFS cases (Welch's two-tailed t-test, p = 3.986E-4).

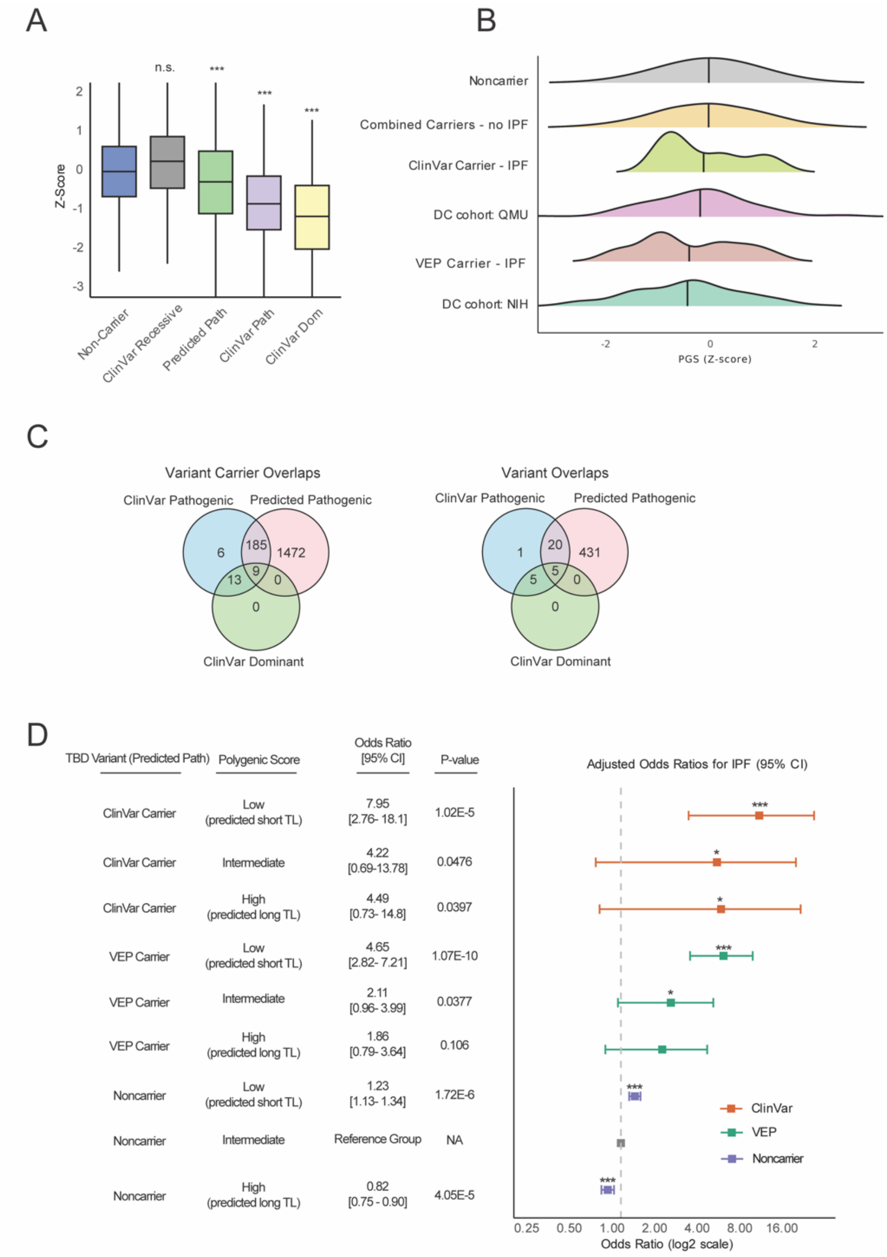

Supplemental Figure S2: Ascertainment strategy and analysis of UK Biobank

A) Measured telomere length in UK Biobank non-carriers and carriers of pathogenic TBD variants, showing carriers of ClinVar mutations in recessive genes (no enrichment for short telomere length) (pairwise t-test with Bonferroni multiple testing correction, alternative = “less”: non-carrier vs Predicted Pathogenic: p=2.53E-20; non-carrier vs ClinVar Pathogenic: p =2.31E-23; non-carrier vs ClinVar Dominant: p = 9.32e-4; non-carrier vs ClinVar Recessive: p = 1).

B) Overlaying PGS distributions for all cohorts including variant carriers with and without IPF.

C) Number within and overlaps between each pathogenic category for variant carriers and individual variants.

D) Odds ratios of idiopathic pulmonary fibrosis in UK Biobank stratified by PGS tertile (top third, middle third, and lowest third) and Clinvar Pathogenic or Predicted Pathogenic variant-carrier status, analyzed separately, using the non-carrier intermediate group as the control group.

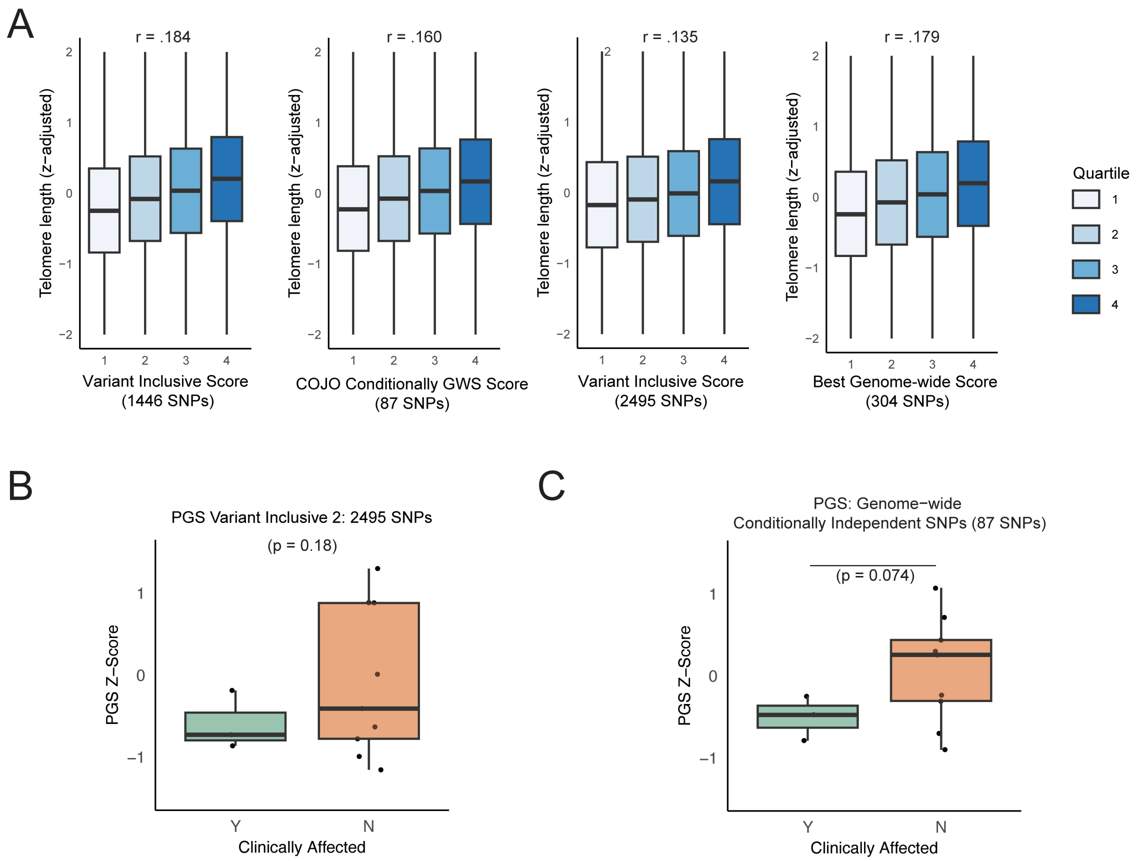

Supplemental Figure S3: Pedigree analysis

A) Measured telomere length by PGS quartile across different PGS scores constructed for pedigree SNPs (Spearman correlation between quartile and adjusted telomere length).

B) Telomere length PGS comparing cases to non-case TERT variant carriers for Variant-inclusive PGS Score 2 (linear mixed model with kinship matrix as random effect, see **Methods**).

C) C) Telomere length PGS comparing cases to non-case TERT variant carriers for COJO Independent Genome-Wide Significant PGS Score (linear mixed model with kinship as random effect).

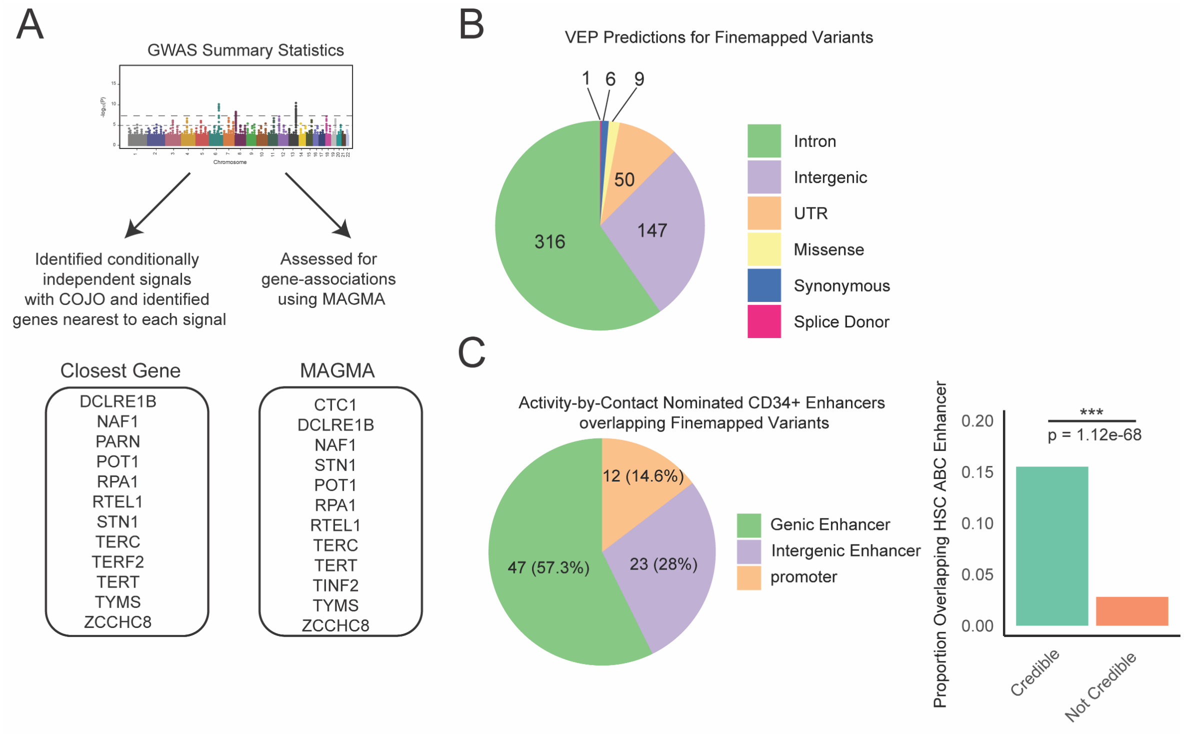

Supplemental Figure S4: Causal gene prioritization and variant functional annotation

A) Strategy to prioritize likely causal genes. Genes closest to conditionally independent genome-wide significant signals were identified. MAGMA was used to identify genes associated with summary statistics.

B) VEP predictions for fine-mapped 90% credible-set variants.

C) Finemapped variants overlapping ABC enhancers in hematopoietic stem cells (enrichment computed using chi-squared test)

Supplemental Methods

UK Biobank

We excluded individuals with mismatches between self-reported and genetically inferred sex, possible sex chromosome aneuploidy, outliers for heterozygosity or missingness, excessive kinship, and individuals who withdrew consent at the time of analysis. Because telomere length has been reported to vary significantly across ancestry groups and our patient population is predominantly white European ancestry, we excluded individuals who were not in the white British ancestry subset defined both by self-report and by genetic ancestry (116).

All of Us

Only samples with matching reported sex and genetically inferred sex were included. Site level QC metrics included QUAL > 60, ExcessHet < 54.69, GQ > 20, DP > 10, AB > 0.2 for heterozygotes. Variants with population-specific allele frequency (AF) > 1% or population-specific allele count (AC) > 100, in any computed ancestry subpopulations were selected. Sites with more than 100 alternate alleles were excluded. A high-quality set of SNPs were used to determine ancestry labels for each sample based on the same labels as gnomAD and were used to filter for European (EUR) population individuals.

National Cancer Institute Inherited Bone Marrow Failure Syndrome samples

Genotype array data was obtained in PLINK file format and imputed using the TOPMed Imputation Server (117–119). The data from the kindred with a shared *TERT* mutation was considered separately from the other TBD cases and only in pedigree-specific analyses.

Queen Mary University of London samples

For the samples of unknown genotype, processed VCF files were imputed using the TOPMed Imputation Server. The resulting FASTQ files from the Azenta sequenced samples were adapter trimmed using cutadapt and then aligned to the hg38 reference genome using bwa-mem. Since the TOPMed Imputation Server is not designed to impute from low-pass sequencing samples, these samples were imputed with GLIMPSE2 using the 1000 Genomes phase 3 release as a reference set (89, 120).

Genome-wide association analysis for telomere length

Genome-wide association study for telomere length

REGENIE was used to conduct a GWAS for telomere length in the UK Biobank (121). For Step 1 of REGENIE, the UKB genotype array files were quality controlled following the recommendations of the REGENIE developers, removing SNPs with minor allele frequency below 1%, minor allele count below 100, genotype missingness greater than 10%, sample missingness greater than 10%, and Hardy-Weinberg equilibrium test p-value exceeding 1e-15.

For Step 2 of REGENIE, we used common SNPs from the November 2023 UK Biobank whole-genome sequencing release (UKB field: 24053). The DRAGEN individual-level variant call files were filtered for minor allele frequency of at least 0.1% using BCFtools and the following quality control criteria: HWE>10e-30, INFO/AN > 0.9*AN, ExcHet >= 0.5 && ExcHet <= 1.5, FILTER="PASS" && MAF>0.001, then merged into PLINK files for REGENIE.

Step 1 of REGENIE was run using recommended parameters with --bsize 1000. Age, sex, and the first ten genetic principal components were included as covariates. Step 2 was run using the .loco and .pred.list files output from step 1 with --quant_traits=true, and age, sex, and the first ten genetic principal components as covariates.

The summary stats were then used with one further processing step: because the qPCR-based telomere length measurement relies on HBB gene amplification as a control, the measurement results in a previously reported artifactual enrichment of genetic associations around the HBB gene (73). Following the procedure previously employed by *Codd et al.*, we removed 1 million bases flanking either end of the HBB gene from the summary stats files before constructing polygenic risk scores.

Polygenic risk score construction using telomere length summary statistics

Primary polygenic risk score construction

To identify the most predictive overall scores in the UK Biobank population, a grid search over linkage disequilibrium thresholds including r^2 values of [0.001, 0.005, 0.01 ,0.02, 0.05, 0.1, 0.2, 0.5, 0.8] and clumping windows including [100, 250, 500, 750, 1000, 1500, 2000, 2500, 3000, 4000, 5000, and 7500] kilobases was performed. For each of these, a range of p-value thresholds was tested using PRSice-2 from 5e-8 to 5e-4 in increments of 1e-7. The most predictive score by variance explained was identified. The following flags were used for PRSice-2: --base-info INFO: 0.6, --beta, --binary-target F, --cov-col "@pc[1-10],sex,age", --model add, using the 5,279,945 SNP set previously defined, and with z-scored log-normalized adjusted telomere length from Codd et al. as the phenotype (74, 77).

From the grid search analysis, there was a range of parameter values that provided similar, greatest prediction accuracy (r^2 0.02, with LD window size 2000 and greater). Of these, the lowest parameter value was selected at which prediction accuracy was observed to plateau (r^2 0.02, with LD window size 2000) for downstream analyses, yielding a polygenic score with 304

genome-wide SNPs (**Table S1)**. The variance explained for telomere length of the best polygenic score using these parameters was 0.071 (**Table S1**).

Comparing polygenic risk scores across cohorts

The SNPs which were included in the best PGS score computed in the UK biobank were used to directly compute scores in each different cohort using PRCise-2 with the following parameters used to avoid following the clumping and thresholding algorithm to just compute scores directly from those SNPs: --no-clump --no-full --no-regress --fastscore. The polygenic score values were then z-scored using the UK Biobank score distribution as a population reference to enable easy interpretation. Thus, the UK Biobank score has a mean of 0 and standard deviation of 1, and all other cohort scores can be interpreted from that reference.

All of Us polygenic risk score construction and testing

Restricting to SNPs genotyped with high quality in All of Us resulted in inclusion of 6,219366 SNPs for score construction. (see "Genotype data from various cohorts") (112). We then re-constructed PGS in the same subset of the UK Biobank as previously with these SNPs, the GWAS summary statistics described above, and the UKB measurement for telomere length used previously. We used the exact same parameters that resulted in the most predictive scores in the UK Biobank in our general analysis (r^2 0.02, with LD window size 2000). This resulted in a 253 SNP PGS (**Table S1**). We then used this score to compute PGS for telomere length in the European (EUR) population in AoU, and in the patients from the NCI cohort. As above these scores were directly computed using PRCise-2 with the following parameters used to avoid following the clumping and thresholding algorithm, to just compute scores directly from those SNPs: --no-clump --no-full --no-regress --fastscore. The polygenic score values were then normalized to the AoU values. Thus, the AoU score has a mean of 0 and standard deviation of 1, and the cohort scores can be interpreted from that reference.

Plotting and comparing score distributions

Score distributions across groups were pairwise compared using Welch's t-test with a two-sided alternate hypothesis. Distributions were plotted with kernel density estimates plotted over boxplots using ggplot2. For KDE plots, bandwidth 1 was used as a parameter.

Ancestry inference

Ancestry inference was performed in KING using the 1000 Genomes phase 3 release data to infer ancestry, based on the AFR, AMR, EAS, EUR, and SAS reference groups (88, 89). KING was run with the following parameters: --pca --projection --rplot. Predominant ancestry was defined as having majority ancestry of one of the five reference groups included.

UK Biobank pathogenic variant carrier analysis

Defining variant sets

ClinVar variants were defined using the following query of the ClinVar database (as of July 11th, 2024):

- (dyskeratosis congenita) OR Coats plus) OR hoyeraal-hreidarsson) OR Revesz syndrome) AND ((("clinsig likely pathogenic"[Properties] or "clinsig likely risk allele"[Properties]) OR ("clinsig pathogenic"[Properties] or "clinsig pathogenic low penetrance"[Properties] or "clinsig established risk allele"[Properties]))) with "Review status" "At least one star" and "Classification type" "Germline", yielding 592 variants.

Pre-processing the UK Biobank whole-exome sequencing data

The UKB exome sequencing final release of 454,787 participants PLINK fileset was filtered for rare variants with allele frequency less than or equal to 1 percent, that passed the recommended variant-level filter requiring that at least 90% of all genotypes for a given variant - independent of variant allele zygosity - are nonmissing and have a read depth of at least 10 (i.e. DP>=10) (122). Variants were further processed to exclude samples with missingness greater than 10%. Indels and SNVs were then extracted by filtering the processed exome files by matching on chromosome, position, reference and alternate alleles, for the variant sets defined above.

Statistical analyses of UKB pathogenic variant carriers

For the comparisons between non-carriers and the different pathogenic carrier sets and for dyskeratosis congenita cases compared to the pathogenic carrier sets, because statistical comparisons were directly between the reference set and the pathogenic sets, pairwise t-tests were used with Bonferroni multiple testing correction. Logistic regression models were fitted to estimate the association between genetic annotations and idiopathic pulmonary fibrosis or aplastic anemia. The models included age, sex, and the first 4 principal components of ancestry as covariates. Odds ratios (ORs) with 95% confidence intervals (CIs) were extracted from each model with pathogenic carrier status as the predictor. Models were fit using the glm function in R with a binomial family, and ORs were obtained by exponentiating the model coefficients.

To test for a PGS contribution to IPF risk in variant-carriers and non-variant carriers with an interpretable odds ratio output, we created variables indicating PGS tertile and carrier status for ClinVar Path variants or Consensus Predicted Path variants (Group 1 = PGS high and carrier, Group 2 = PGS high and non-carrier, Group 3 = PGS intermediate and carrier, etc.). For each group, UKB participants were coded as 1 if part of this group, as 0 if not a member of the other pathogenic variant groups, and as NA if included in the other variant carrier groups. We then

performed logistic regression adjusting for age, sex and the first 4 PCs of ancestry, with IPF as the dependent variable.

To test for an interaction between the PGS and rare variant status, we performed a similar logistic regression with age, sex, the first 4 PCs, the individual PGS values (non-binned), and ClinVar variant carrier status, and an interaction term between PGS and variant carrier status.

To conduct the mediation analysis, we used the mediator package in R (99). The mediator was z-score adjusted telomere length, and the treatment variable was the PGS. Following a standard mediation analysis, a logistic regression was conducted regressing IPF on the PGS with telomere length, sex, age, and presence of a TBD variant as independent variables. A linear regression was also run regressing telomere length on the PGS with sex, age, and presence of a TBD variant as independent variables. The *mediator* function was then used with these two models as inputs to examine the extent to which the total effect of the PGS exposure on the IPF outcome operates through measured average telomere length.

Analysis of expressivity within a family

Pedigree analysis polygenic risk score construction

We utilized the same set of 5,279,945 SNPs to compute polygenic risk scores in the pedigree cohort. We constructed four different polygenic scores. Three were constructed taking the best score using clumping and thresholding with various sets of hyperparameters. For all scores, the LD r^2 threshold and window size for calculating LD was set, and then scores of increasing p-value were tested (starting from 5E-8, and increasing at intervals of 5E-7), and the score with the best explanatory power in the UK Biobank was selected. The following sets of clumping and thresholding hyperparameters were used: “PGS: Variant Inclusive 1”, we used an LD r^2

threshold of 0.2 and an LD window size of 2000 kb, resulting in inclusion of 1446 SNPs and then calculated the best score (**Table S1**). For “PGS: Variant Inclusive 2”, we used an LD r^2 threshold of 0.5 and an LD window size of 500 kb, resulting in inclusion of 2495 SNPs (**Table S1**). For the PGS: Best Genome-wide Conditionally Significant SNP Score, we included all conditionally significant independent non-ambiguous signals using GCTA-COJO (see Methods: *Finemapping*), resulting in inclusion of 87 SNPs (**Table S1**). Finally, we used the same parameters as our best genome-wide score, with an LD r^2 threshold of 0.02, and an LD window size of 2000 kb, including 304 SNPs as described previously.

Analysis of convergence of common and rare variation

Gene prioritization

From the telomere length GWAS summary statistics, genes were prioritized using two approaches. MAGMA was used with parameters --gene-model snp-wise=mean. A false-discovery rate of 0.01 was applied to identify MAGMA prioritized genes (123). For closest gene analysis, the COJO conditionally genome-wide significant SNPs were used and the three nearest genes were identified based on location (overlapping or nearest distance to start or end) using BEDtools (124). The MAGMA and closest gene prioritized sets were combined into a single common-variation prioritized set **(Figure S4A)**. A hypergeometric test was used to test for enrichment of the known TBD causal genes in this set.

Conditional and joint analysis using summary data

Conditionally independent SNP signals were identified using GCTA-COJO with the telomere length summary statistics, using the UK Biobank dataset WGS PLINK fileset as a reference with the following parameters: --cojo-wind 10000 --cojo-slct --cojo-p 5e-8 (125).

Finemapping

Finemapping was performed using FINEMAP (126). Sentinel SNPs for finemapping were selected by using COJO outputs and iteratively selecting COJO independent signals with a p-value less than 5e-6, and then iteratively expanding the finemapping region if other COJO signals were found within one megabase of each signal. For each sentinel region, all SNPs were extracted from the UKB WGS PLINK files and LD matrices computed using plink with parameter --r square. These sentinel regions were then used to run FINEMAP with the following parameters: --sss --n-causal-snps 10.

Supplemental Note

Mediation analysis

We use a mediation analysis framework (99) to address the extent to which the causal relationship between the TL PGS and IPF operates through mean telomere length, controlling for age, sex, and the presence of a pathogenic variant (**Supplemental Methods**). We find that approximately 37% of the total effect of the PGS on IPF outcomes is mediated through effects on mean telomere length (**Table S6**). This provides strong evidence that the TL PGS captures common genetic effects of telomere length influencing IPF. While this is a substantial fraction, this result could indicate that the relationship is complex and that the PGS also influences IPF outcomes through other causal mechanisms, or that our telomere length measures fail to appropriately capture cell-type- or chromosome- specific telomere length. We hypothesize that mean telomere length is only a proxy for the causal disease process that is most directly related to telomere length and maintenance preventing accumulation of extremely short telomeres, and that the TL PGS may capture some of these effects beyond its direct role in predicting mean telomere length.

Supplemental Table

**Table S1:** PGS Model Summary							
	**Number of SNPs**	**Threshold**	**Full.R2**	**Null. R2**	**PRS.R2**	**Coefficient**	**Standard Error**
UKB Main Analysis	304	3.61E-05	0.070672	0.04194	0.028732	442.626	6.99436
All of Us SNPs only	253	1.77E-05	0.07038	0.04194	0.02844	380.87	6.05029
**Scores used in pedigree analysis**							
Pedigree Variant Inclusive PGS Score 1	1446	0.000448	0.065896	0.04194	0.023957	779.79	13.5292
Pedigree Variant Inclusive PGS Score 2	2495	0.014786	0.056726	0.04194	0.014786	413.661	9.18013
COJO Genome-wide Significant Score: all COJO genome-wide significant signals used in score							
COJO Genome-wide significant Score	87	5.00E-08					

Table S2: SNPs in PGS
See Excel file

**Table S3:** Genes Included in Analyses		
**Gene**	**Mode of inheritance**	**Included/excluded**
NAF1	Dominant	Included
RPA1	Dominant	Included
TERC	Dominant	Included
TINF2	Dominant	Included
ZCCHC8	Dominant	Included
ACD	Dominant or recessive	Included
RTEL1	Dominant or recessive	Included
TERT	Dominant or recessive	Included
DKC1	X-linked recessive	Male carriers included
CTC1	Recessive	Excluded
NHP2	Recessive	Excluded
NOP10	Recessive	Excluded
POT1	Recessive	Excluded
STN1	Recessive	Excluded
WRAP53	Recessive	Excluded
DCLRE1B	Recessive	Excluded
PARN	Recessive	Excluded

**Table S4:** VEP Carriers per Gene	
**Total carriers per gene**	
RTEL1	836
ACD	239
ZCCHC8	247
TERT	179
TINF2	140
DKC1	25

**Table S5:** VEP Total unique variants per gene	
RTEL1	252
TERT	93
ZCCHC8	92
ACD	66
TINF2	56
DKC1	5

Table S6: VEP Distinct Variants and # Carriers
See Excel file.

**Table S7:** ClinVar Pathogenic Total carriers per gene	
RTEL1|RTEL1-TNFRSF6B	190
TERT	14
DKC1	8
ZCCHC8	1

**Table S8:** ClinVar Pathogenic Total unique variants per gene	
RTEL1|RTEL1-TNFRSF6B	20
TERT	9
DKC1	1
ZCCHC8	1

Table S9: Distinct variants with total carriers		
**Gene**	**ID**	**n**
DKC1	23:154773152:C:T	8
RTEL1-TNFRSF6B|RTEL1	20:63689813:C:T	2
RTEL1|RTEL1-TNFRSF6B	20:63659463:C:T	2
RTEL1|RTEL1-TNFRSF6B	20:63661385:C:T	2
RTEL1|RTEL1-TNFRSF6B	20:63662520:C:T	29
RTEL1|RTEL1-TNFRSF6B	20:63662876:C:A	2
RTEL1|RTEL1-TNFRSF6B	20:63678347:G:T	3
RTEL1|RTEL1-TNFRSF6B	20:63679947:G:A	1
RTEL1|RTEL1-TNFRSF6B	20:63687936:G:A	7
RTEL1|RTEL1-TNFRSF6B	20:63689628:C:T	1
RTEL1|RTEL1-TNFRSF6B	20:63689866:G:T	1
RTEL1|RTEL1-TNFRSF6B	20:63690172:C:T	11
RTEL1|RTEL1-TNFRSF6B	20:63690205:C:T	2
RTEL1|RTEL1-TNFRSF6B	20:63691799:C:T	2
RTEL1|RTEL1-TNFRSF6B	20:63692826:C:T	1
RTEL1|RTEL1-TNFRSF6B	20:63693004:G:T	2
RTEL1|RTEL1-TNFRSF6B	20:63693211:C:T	57
RTEL1|RTEL1-TNFRSF6B	20:63693247:C:T	34
RTEL1|RTEL1-TNFRSF6B	20:63694739:A:C	24
RTEL1|RTEL1-TNFRSF6B	20:63695223:T:C	1
RTEL1|RTEL1-TNFRSF6B	20:63695619:G:A	6
TERT	5:1254476:C:T	2
TERT	5:1258604:G:A	2
TERT	5:1264435:G:A	1
TERT	5:1266524:C:T	3
TERT	5:1272247:G:A	1
TERT	5:1278640:C:T	2
TERT	5:1279316:G:A	1
TERT	5:1282498:G:A	1
TERT	5:1294198:G:A	1
ZCCHC8	12:122483508:G:A	1

**Table S10:** ClinVar Dominant-Acting Total carriers per gene	
TERT	14
DKC 1	8

**Table S11:** ClinVar Dominant-Acting Total unique variants per gene	
TERT	9
DKC1	1

**Table S12:** ClinVar Dominant-Acting Distinct variants with total carriers		
Gene	ID	n
DKC1	23:154773152:C:T	8
TERT	5:1254476:C:T	2
TERT	5:1258604:G:A	2
TERT	5:1264435:G:A	1
TERT	5:1266524:C:T	3
TERT	5:1272247:G:A	1
TERT	5:1278640:C:T	2
TERT	5:1279316:G:A	1
TERT	5:1282498:G:A	1
TERT	5:1294198:G:A	1

Table S13: IPF regression with interaction term between rare variant status and PGS

term	estimate	std.error	statistic	p.value	conf.low	conf.high	OR	lower	upper
(Intercept)	-16.986317	1.66356975	-10.210763	1.77E-24	-20.248652	-13.727209	4.20E-08	1.61E-09	1.09E-06
age	0.1175038	0.00323927	36.2748109	4.04E-288	0.1112018	0.12390244	1.12468591	1.11762042	1.13190544
sex	0.55651568	0.03790615	14.6814098	8.48E-49	0.48240352	0.63101794	1.74458322	1.61996334	1.87952285
pc1	-0.0626089	0.03190376	-1.9624306	0.04971237	-0.1251568	-8.94E-05	0.93931075	0.88235857	0.99991065
pc2	0.07848167	0.02727489	2.87743328	0.00400925	0.02502946	0.13195094	1.08164352	1.02534533	1.14105233
pc3	-0.0003111	0.02304746	-0.013498	0.98923048	-0.0454923	0.04485715	0.99968895	0.95552691	1.04587844
pc4	0.03556102	0.02446509	1.45354135	0.14607351	-0.0123955	0.08351129	1.03620088	0.98768097	1.08709749
clinvar_pathogenic_exclusiveTRUE	1.76999844	0.34894679	5.07240215	3.93E-07	1.00821184	2.39383209	5.87084421	2.74069583	10.9553957
prs	-0.197875	0.01847408	-10.710953	9.04E-27	-0.2340746	-0.1616536	0.82047244	0.79130278	0.85073587
clinvar_pathogenic_exclusiveTRUE:prs	0.19716932	0.33781389	0.58366255	0.55944737	-0.4869417	0.85378983	1.21795025	0.61450286	2.34853054

Table S14: IPF regression: no interaction term between rare variant status and PGS

term	estimate	std.error	statistic	p.value	conf.low	conf.high	OR	lower	upper
(Intercept)	-16.609698	1.66437772	-9.9795246	1.87E-23	-19.873597	-13.348993	6.12E-08	2.34E-09	1.59E-06
age	0.11750884	0.00323916	36.2775723	3.65E-288	0.11120705	0.12390724	1.12469157	1.11762629	1.13191087
sex	0.55638463	0.03790366	14.6789165	8.80E-49	0.48227744	0.63088198	1.74435461	1.61975911	1.87926732
pc1	-0.0629116	0.03190203	-1.9720257	0.04860667	-0.1254559	-0.0003954	0.93902646	0.88209469	0.9996047
pc2	0.07883957	0.02727494	2.89054999	0.00384568	0.02538713	0.13230872	1.08203072	1.02571213	1.14146066
pc3	-0.0010693	0.02304782	-0.0463935	0.96299661	-0.0462512	0.04409965	0.9989313	0.9548021	1.04508649
pc4	0.03534061	0.02446246	1.4446873	0.14854574	-0.0126109	0.08328557	1.03597251	0.98746833	1.08685214
clinvar_pathogenic_exclusiveTRUE	1.71873551	0.34615357	4.96523981	6.86E-07	0.96567501	2.33863795	5.57747133	2.62656002	10.3671064
prs_quintiles	-0.2038277	0.02289284	-8.903559	5.41E-19	-0.2487701	-0.1590198	0.81560285	0.77975921	0.85297951

Table S15: IPF regression in rare variant carriers only

term	estimate	std.error	statistic	p.value	conf.low	conf.high	OR	lower	upper
(Intercept)	-29.218271	15.5360899	-1.8806709	0.06001671	-59.809686	1.16568389	2.04E-13	1.06E-26	3.20811615
age	0.08153704	0.02625166	3.10597673	0.00189652	0.03264826	0.13612642	1.0849534	1.03318707	1.14582674
sex	0.65233942	0.35438116	1.8407847	0.06565312	-0.0280422	1.37400247	1.92002733	0.97234737	3.95113337
pc1	-0.2318008	0.31248565	-0.7417965	0.45821063	-0.8423954	0.38323483	0.79310412	0.43067766	1.46702248
pc2	0.30583446	0.25720944	1.18904833	0.23442065	-0.1976317	0.81207125	1.35775752	0.82067209	2.2525688
pc3	-0.1508198	0.20784093	-0.72565	0.46805334	-0.5581866	0.25793888	0.86000267	0.57224581	1.29425971
pc4	0.56417509	0.22157628	2.54618897	0.01089062	0.13188825	1.00255956	1.757997	1.14098081	2.72524833
prs	-0.2688569	0.16735185	-1.6065366	0.10815606	-0.5995345	0.05756177	0.76425264	0.54906717	1.0592507

**Table S16:** Mediation Analysis Outputs			
Effect	Estimate	Lower 95% CI	Upper 95% CI
CDE	0.884881	0.852347	0.918657
NDE	0.884881	0.852347	0.918657
NIE	0.924554	0.917598	0.931563
Total Effect	0.818121	0.788559	0.848791
Proportion Mediated	0.367059	NA	NA

**Table S17:** PGS Difference by Sex				
**NCI**				
	**N**	**mean_pgs (z-score)**	**sd_pgs**	**p_val**
F	36	-0.521416	1.024784	0.583266
M	54	-0.317239	1.006613	
NA	2	-2.314383	0.158883	
**DCR**				
	**N**	**mean_pgs (z-score)**	**sd_pgs**	**p_val**
F	43	-0.12608	0.929345	0.354072
M	147	-0.217945	1.0651	
**UKB**				
	**N**	**mean_pgs (z-score)**	**sd_pgs**	**p_val**
F	220471	-0.03172	1.000117	0.972971
M	187366	-0.031827	0.995346	
**AoU**				
	**N**	mean_pgs (z-score)	sd_pgs	p_val
F	74529	-0.000711	1.0001	0.6304
M	51017	0.002054	1.0002	
NA	2650	-0.019569	0.9938	

**Table S18:** Ancestry			
		**NCI**	
			**N**
		AFR	1
		AMR	24
		EAS	4
		EUR	62
		SAS	1
		**DCR**	
			**N**
		AFR	3
		AMR	26
		EAS	7
		EUR	139
		SAS	15
		**UKB**	
			**N**
		AFR	9037
		AMR	64846
		EAS	2711
		EUR	403505
		SAS	10442
		**AoU**	
			**N**
		EUR	128196

**Table S19:** Measured telomere length				
**NCI**				
	**Population Z-score**		**length percentile**	**N**
Min	-7.845782		<=1%	59
25%	-3.405093		1–10%	16
Median	-1.639583		>10%	12
75%	-0.542015		NA	5
Max	1.792239			
**DCR**				
	**length percentile**	**N**		
	>10%	45		
	<=10%	36		
	<=1%	75		
	NA	58		
**UKB**				
	**population z-score**			
Min	-15.2824			
25%	-0.671664			
Median	-0.02959			
75%	0.612331			
Max	12.1395			
**AoU**				
not measured.				
